# Activated carbon-coated iron oxide magnetic nanocomposite (IONPs@CtAC) loaded with morin hydrate for drug-delivery applications

**DOI:** 10.3389/fchem.2024.1477724

**Published:** 2024-10-21

**Authors:** Yusuf Doğan, Cem Öziç, Erdal Ertaş, Ayşe Baran, Gvozden Rosic, Dragica Selakovic, Aziz Eftekhari

**Affiliations:** ^1^ Kızıltepe Vocational School, Mardin Artuklu University, Mardin, Türkiye; ^2^ Department of Basic Medical Sciences, Department of Medical Biology, Faculty of Medicine, Kafkas University, Kars, Türkiye; ^3^ Department of Food Technology, Vocational School of Technical Sciences, Batman University, Batman, Türkiye; ^4^ Department of Biology, Graduate Education Institute, Mardin Artuklu University, Mardin, Türkiye; ^5^ Department of Physiology, Faculty of Medical Sciences, University of Kragujevac, Kragujevac, Serbia; ^6^ Department of Biochemistry, Faculty of Science, Ege University, Izmir, Türkiye; ^7^ Department of Life Sciences, Western Caspian University, Baku, Azerbaijan

**Keywords:** anticancer activity, magnetic nanocomposite, morin hydrate, nanodrug-delivery system, *Celtis tournefortii*, activated carbon

## Abstract

Cancer is a major disease that affects millions of people around the world every year. It affects individuals of all ages, races, and backgrounds. Since drugs used to treat cancer cannot distinguish between cancerous and healthy cells, they cause systemic toxicity along with serious side effects. Recently, controlled drug-release systems have been developed to reduce the side effects caused by anticancer drugs used for treatment. Morin is an anticancer drug with a flavonol structure. It has been extensively researched for its antioxidant, anti-inflammatory, antitumoral, and antibacterial properties, especially found in Chinese herbs and fruits, and its multiple positive effects on different diseases. In this study, a nanocomposite with magnetic properties was synthesized by coating biocompatible activated carbon obtained using the fruits of the *Celtis tournefortii* plant on the surface of iron oxide magnetic nanoparticles. Characterization of the synthesized activated carbon-coated iron oxide magnetic nanocomposite was confirmed by Fourier transform infrared, scanning electron microscopy, energy-dispersive X-ray spectrometry, X-ray diffraction, dynamic light scattering, zeta potential, and vibrating sample magnetometry. The cytotoxic effects of the drug-loaded magnetic nanocomposite were examined in HT-29 (colorectal), T98-G (glioblastoma) cancer cell lines, and human umbilical vein endothelial cell (HUVEC) healthy cell line. The morin loading and release behavior of the activated carbon-coated iron oxide magnetic nanocomposite were studied, and the results showed that up to 60% of the adsorbed morin was released within 4 h. In summary, activated carbon-coated iron oxide magnetic nanocomposite carriers have shown promising results for the delivery of the morin drug.

## 1 Introduction

The emergence of new therapeutic options through the utilization of targeted pharmaceuticals, contingent upon the synthesis and formulation delivery of novel compounds derived from various sources, including plant extracts, is a promising advancement (Montazersaheb et al., 2023; Ullah et al., 2024; Aliyeva, 2024). In order to reduce the side effects caused by drugs used in cancer treatment researchers are working on systems that allow drugs to directly reach the targeted organ in human body. The success of chemotherapy, one of the treatment options for cancer, varies depending on the type of drug used, how it is delivered to the disease area, and its dosage (Włodarczyk et al., 2022; Huda et al., 2020).

Nowadays, iron oxide magnetic nanoparticles (IONPs) have attracted great attention due to their potential biomedical applications such as immunoassay (Stueber et al., 2021), magnetic resonance imaging (Chen et al., 2023), bioseparators (Shabatina et al., 2020), and targeted drug delivery by applying an external magnetic field (Tarhan et al., 2023). At the same time, IONPs have desirable characteristics, including biocompatibility (Feng et al., 2024) and low toxicity (Khan et al., 2020), when coated with an appropriate material. The IONP core acts as a carrier for magnetic targeting, while activated carbon (AC) on IONPs offers sites for further modifications (Rehman et al., 2023). This activated carbon can bind to IONPs through adsorption or covalent bonding (IONPs–AC) and combine with the anti-cancer drug morin (MR) through the functional groups in its structure.

Morin (3,5,7,2′,4′-pentahydroxyflavone) is a member of the flavonoid groups reported to be an effective chemotherapeutic agent and an important agent used in cancer treatment (Alonso et al., 2022). Additionally, numerous reports suggest that morin has a wide range of therapeutic applications, such as anti-inflammatory (Liu et al., 2021), antioxidant (Ding et al., 2020), inducing apoptosis in a hepatocellular carcinogenesis model (Mottaghi and Abbaszadeh, 2021), and xanthine oxidase inhibition activity (Balaga et al., 2023).

In this study, IONP magnetic nanoparticles were produced by the co-precipitation method and coated with activated carbon obtained from the *Celtis tournefortii* (Ct) plant. Then, this IONPs@CtAC magnetic nanocomposite was used as the drug carrier. Fourier transform infrared (FTIR), scanning electron microscopy (SEM), energy-dispersive X-ray spectrometry (EDX), dynamic light scattering (DLS), zeta potential, and vibrating sample magnetometry (VSM) techniques were used to characterize the synthesized magnetic nanocomposite. MR drug loading and *in vitro* drug release profiles were examined in HT-29 (colorectal), T98-G (glioblastoma) cancer cell lines, and human umbilical vein endothelial cell (HUVEC) healthy cell line.

## 2 Materials and methods

### 2.1 Chemicals and reagents

The materials iron(III) chloride hexahydrate (≥99% purity), iron(II) chloride tetrahydrate (≥99% purity), (37%, ACS reagent), sodium hydroxide (≥97.0%, pellets), ammonium hydroxide (≥28% purity), morin hydrate (≥100% purity), methanol (≥99.8%, ACS reagent), ethanol (96%, ACS reagent), and zinc chloride (≥98% purity) were purchased from Sigma-Aldrich. All other chemicals used were of analytical grade. Double-distilled water was used for synthesis, solution preparation, and other purposes throughout the experiment.

### 2.2 Characterization

The synthesized CtAC, IONPs@CtAC, and IONPs@CtAC-MR were characterized to determine their structural characteristics and morphology using the following methods:

Fourier transform infrared spectrophotometry (FT-IR): functional groups present in IONPs@CtAC and IONPs@CtAC-MR were analyzed using an Agilent Cary 630 FTIR spectrometer.

Scanning electron microscopy (SEM): the surface morphology of magnetic nanocomposites IONPs@CtAC and IONPs@CtAC-MR was analyzed using a QUANTA 400F device operating at a voltage range of 200 V to 30 kV. To obtain SEM images, 20 kV was used from values ranging from 200 V to 30 kV. No contrast agent was used while taking the images. The natural states of the materials were used.

Energy-dispersive X-ray spectroscopy (EDX): elemental mapping was performed using a JEOL 210 F microscope.

Zeta potential: stability of nanoparticles in the solution was measured using a Zetasizer Nano ZS from Malvern Instruments Ltd. at 25°C.

Dynamic light scattering: particle agglomerate size distributions were evaluated using a Mastersizer 2000 from Malvern.

X-ray diffraction (XRD): XRD patterns for the IONPs@CtAC and IONPs@CtAC-MR were performed using a Rigaku RadB-Dmax II at 2θ = 3°–80° with a scan rate of 1°/min.

Vibrating sample magnetometer (VSM): the magnetic properties of IONPs@CtAC were observed at 25°C using a VSM (Quantum Design PPMS-9T).

UV–visible spectrophotometry: the UV–visible absorption spectrum was recorded using an Agilent Cary 60 spectrophotometer.

Before taking measurements, the samples were obtained from the stock solution by diluting them with pure water as required.

### 2.3 Synthesis of CtAC

In the process of synthesizing activated carbon (AC), the fruit part and the hard inner part of the *C. tournefortii* (Ct) plant were subjected to initial washing and drying steps to eliminate foreign contaminants. The fruits were pulverized to increase the activation surface area. Ten grams of the powder sample obtained by grinding the leaves of the *C. tournefortii* plant after drying was weighed and placed in a 500-mL Erlenmeyer flask, and then, a solution of zinc chloride with a concentration of 1 M (for activation of activated carbon) was added and mixed in a shaking water bath at 90°C for 1 hour (Zhao et al., 2022).

Following cooling to ambient temperature, the mixed precipitate was homogeneously put in a 500-mL glass crystallization container and dried in an oven at 110^°^C for 24 h. Then, the dried mixture was transferred to porcelain crucibles under room conditions and carbonized in a muffle furnace at 550^°^C for 4 h, and thus, activated carbon was synthesized using *Celtis tournefortii* (Dardagan) fruits. Carbonization increases the surface area by creating pores in the carbon structure. The HCl solution was washed several times with 0.1 N to remove the remaining ions that did not react on the surface of CtAC. After washing the HCl solution, the composite was washed with distilled water and left to dry in an oven set at 75^°^C (Hamat et al., 2024; Maniom and Gunasegaran, 2023).

### 2.4 Synthesis of the IONPs@CtAC magnetic nanocomposite

**FIGURE 1:**
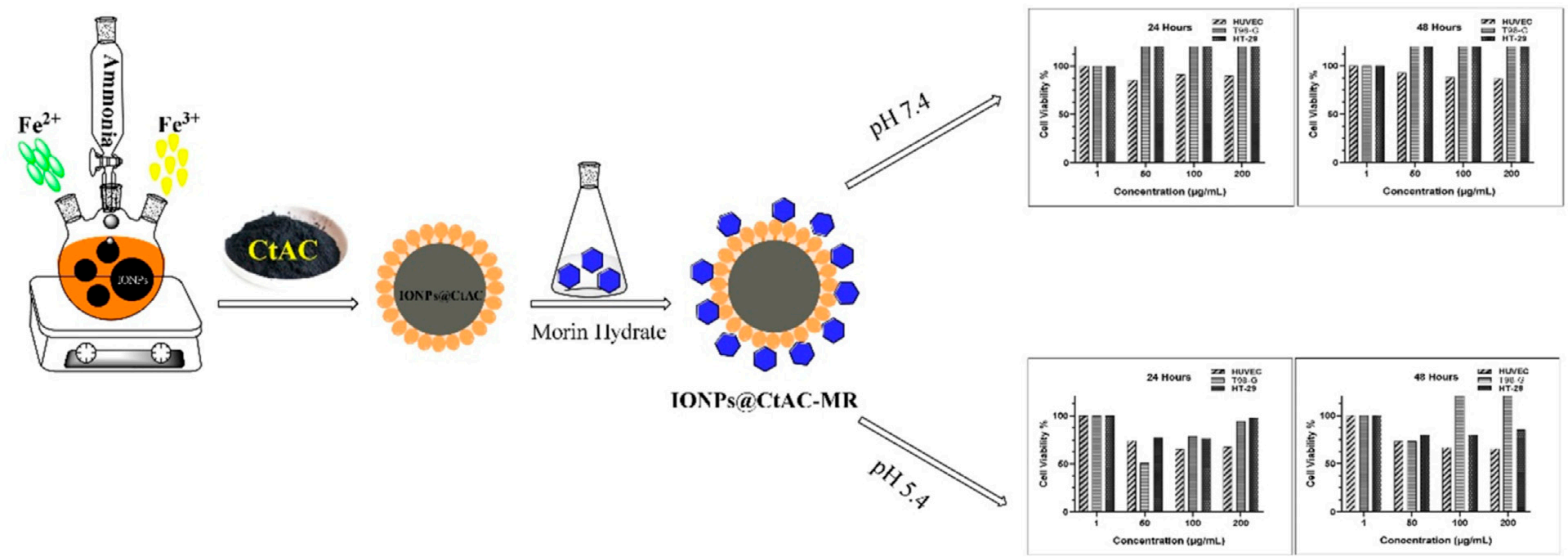
Synthesis mechanism of the IONPs@CtAC magnetic nanocomposite.

IONPs@CtAC was synthesized by the co-precipitation method, which is a simple and useful approach, with some modifications, as stated in the literature (Duan et al., 2020). After 6.0 g of iron (III) hexahydrate was dissolved in 100 mL of distilled water, a few drops of concentrated HCl solution were added to the solution to avoid hydrolysis of iron (III) ions in the solution environment. In the next step, 4 g of iron (II) tetrahydrate was added to the solution, and the temperature of the solution mixture was slowly increased to 90°C. This process continued for 30 min, and the solution was mixed. At the end of 30 min, 10 mL of 28% ammonia solution was added to the mixture, and stirring was continued for another 30 min at this temperature until the solution turned black. In the next step, the solution prepared by adding 0.50 g of CtAC in 50 mL of water was added to the mixture. One hour after the addition of CtAC, the system was turned off in a shaking water bath, and the IONPs@CtAC obtained were allowed to cool at room temperature. The synthesized IONPs@CtAC nanocomposite were separated from the mixture with an external magnet, washed, and dried in a desiccator at room temperature (Inbaraj et al., 2021; Kyzas et al., 2022). To remove the substances that did not react with IONPs@CtAC and remained in the environment, the IONPs@CtAC nanocomposite was washed 4–5 times with distilled water and then left to dry in the oven (set at 75°C). The IONPs@CtAC nanocomposite was stored in a dark environment for use in other studies. The synthesis mechanism of IONPs@CtAC magnetic nanocomposite is shown in Figure 1.

### 2.5 Loading and release studies of morin (MR)

Loading of MR into the IONPs@CtAC magnetic nanocomposite was carried out by adding 100 μg/mL MR solution dissolved in methanol onto 50 mg of the nanocomposite, stirring at 200 rpm at room temperature for 24 h, and then measuring the unbound portion using UV–vis spectrophotometry according to the formula below. Subsequently, the particles were isolated from the solution using an external magnet based on their magnetic properties, and the concentration of MR in the solution was measured at a wavelength of 385 nm. The drug loading was determined by subtracting the initial concentration of MR from the concentration of MR in the supernatant. Afterward, the magnetic nanoparticles that contained the drug were separated using a magnetic field and then underwent a drying process.

$$\text{Drug loading efficiency }\left(\%\right)=\frac{\text{Total}\;\text{amount}\;\text{of}\;\text{drug}-\text{Free}\;\text{drug}\;\text{in}\;\text{the}\;\text{supernatant}\;}{\text{Total}\;\text{amount}\;\text{of}\;\text{drug}}\times 100.$$

The MR release study involved analyzing 10 mg of dried drug-loaded nanoparticles in 5 mL of PBS (pH 5.4 and 7.4) at a temperature of 37°C, while stirring for durations of 1, 3, 6, 12, 24, 48, and 72 h. After the designated incubation periods, samples were collected, and the quantity of medication released was assessed using UV–vis spectrophotometry at a wavelength of 385 nm. Drug release studies were carried out by measuring according to the following formula:

$$\text{Drug release }\left(\%\right)=\frac{\text{Released}\;\text{drug}}{\text{Total}\;\text{drug}}\times 100.$$

### 2.6 Cell culture

The cell viability test was carried out at the Dicle University Faculty of Veterinary Medicine Cell Culture Laboratory. The healthy (HUVEC) and cancer cell lines (T98-G and HT-29) were provided from the “American Type Culture Collection” (ATCC). The selected cell lines were propagated in a cell culture medium in a T75 flask, as reported by Baran et al., and incubated at 37°C in a 5% CO_2_ environment (Baran et al., 2023). When the cells reached 80%–90% confluency, they were taken from the flasks, and cell numbers were determined by the hemocytometric method. The cells whose numbers were determined were inoculated into 96-well plates in 10 × 10^5^ numbers in three replicates in 90 µL medium in each well and in microplates in duplicate for pH 5.4 and pH 7.4 (to perform two separate time applications, 24 and 48 h). The cells were left undisturbed for 24 h to adhere to the microplate’s bottom. The next day, were seeded plates at various concentrations (200, 100, and 50 μg/mL). Liquids prepared with PBS buffer at pH 5.4 and pH 7.4. Were applied to the cells in the control group.

At 24 and 48 h after the application, an MTT test was performed to determine the changes in cell viability. Ten microliters of the prepared MTT solution (5 mg/mL) was added to each well containing cells in the microplate and incubated for 3 h at 37°C in a humid environment containing 5% CO_2_. After 3 h, the medium was removed, and 100 μL of DMSO was added to each well. After the microplates were left in the shaker for 20 min, the optical density (OD) values in the wells were determined with a UV–vis spectrophotometer (Multi Scan Go, Thermo) (Ipek and Baran, 2024).

The average absorbance values obtained by reading the control wells were accepted as 100% live cell values. The absorbance values obtained from pH 5.4 and pH 7.4 applied wells were accepted as % viability by proportioning them to the control absorbance value. MTT trials were repeated three times on different days.

### 2.7 Statistical analysis

The data obtained from the study were analyzed in the IBM SPSS 21.0 package program. Data were compared between groups using unpaired t-test and among multiple groups by one-way ANOVA, followed by Tukey’s *post hoc* tests. *P* < 0.05 was considered statistically significant.

## 3 Results and discussion

### 3.1 FTIR studies

**FIGURE 2:**
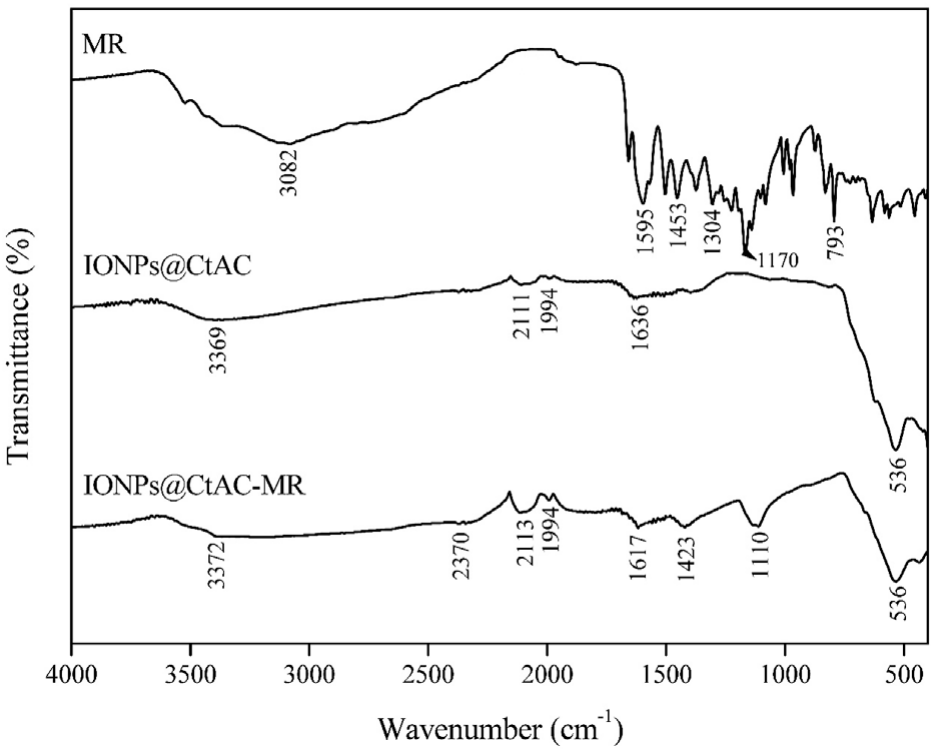
FTIR spectrum comparison of MR, IONPs@CtAC, and IONPs@CtAC-MR.

FT-IR spectra of MR, IONPs@CtAC, and IONPs@CtAC-MR are shown in Figure 2. The 3,369 and 3,372 cm^−1^ peaks of IONPs@CtAC and IONPs@CtAC-MR can be assigned to the O–H bond due to the hydroxyl on the surface of the structure. When the spectra of IONPs@CtAC and IONPs@CtAC-MR are examined, the peak at 2,111 cm^−1^ corresponds to the C≡C stretching of alkynes (Emenike et al., 2023); when the spectra of IONPs@CtAC and IONPs@CtAC-MR nanocomposites at 2,113 cm^−1^ are examined, the measured peak is attributed to the presence of C–H and alkyne groups (Xavier, 2021; Ferreira et al., 2017), and the peaks observed at 1,990–1,994 cm^−1^ indicate stretching vibration of C=C bonds (in alkynes) (Al and Kabakcı, 2024). The peaks seen at 1,617 and 1,636 cm^-1^ (C=O group) correspond to the presence of hydroxyl and carboxylic acid groups (Al-Musawi et al., 2024). The spectra of IONPs@CtAC and IONPs@CtAC-MR are given in Figure 2, and the peak seen at 536 cm^−1^ indicates the presence of Fe–O bond (Tural et al., 2024). Additionally, the peak at 1,170 cm^−1^ signifies the presence of the (C–C–O) group resulting from the bonding of MR (Cunha et al., 2023). As seen in Figure 2, when the FTIR spectrum of the MR drug is examined, the peak seen at 3,082 cm^−1^ indicates the presence of hydroxyl groups. Moreover, the peak originating from the carbonyl group (C=O stretching vibration) is at 1,658 cm^−1^. The C=C stretching vibrations specific to the aromatic rings in the structure of MR are seen at 1,595 and 1,453 cm^−1^. The C–O–C stretching vibrations (ether group) are located at 1,304, 1,226, and 1,170 cm^−1^ (De Gaetano et al., 2023).

### 3.2 SEM analysis

**FIGURE 3:**
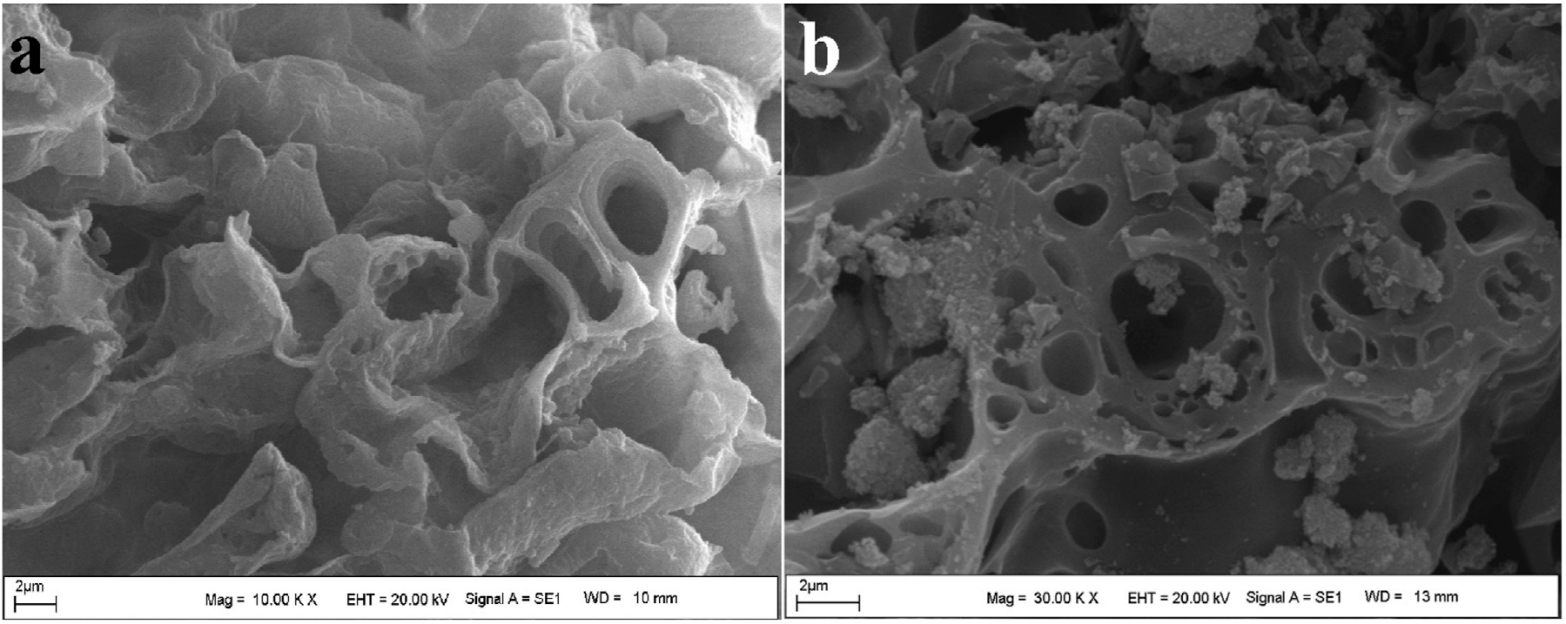
SEM images of **(A)** IONPs@CtAC and **(B)** IONPs@CtAC-MR.

SEM images were used to obtain more information about the surface morphology of morin before and after loading onto the IONPs@CtAC magnetic nanocomposites. Figures 3A, B show the SEM images of IONPs@CtAC and IONPs@CtAC-MR, respectively, and porous structures are observed in both IONPs@CtAC and IONPs@CtAC-MR. When the IONPs@CtAC-MR image is examined, it can be seen that MR is loaded in the porous structures and non-porous parts (Vinayagam et al., 2022; Hajighasemkhan et al., 2022). In addition, porous structures are formed less than they should be because IONPs@CtAC and IONPs@CtAC-MR magnetic nanocomposites agglomerate and come together due to drying. This aggregation and coming together also affects the loading status of the MR (Hussein-Al-Ali et al., 2021).

### 3.3 EDX analysis

**FIGURE 4:**
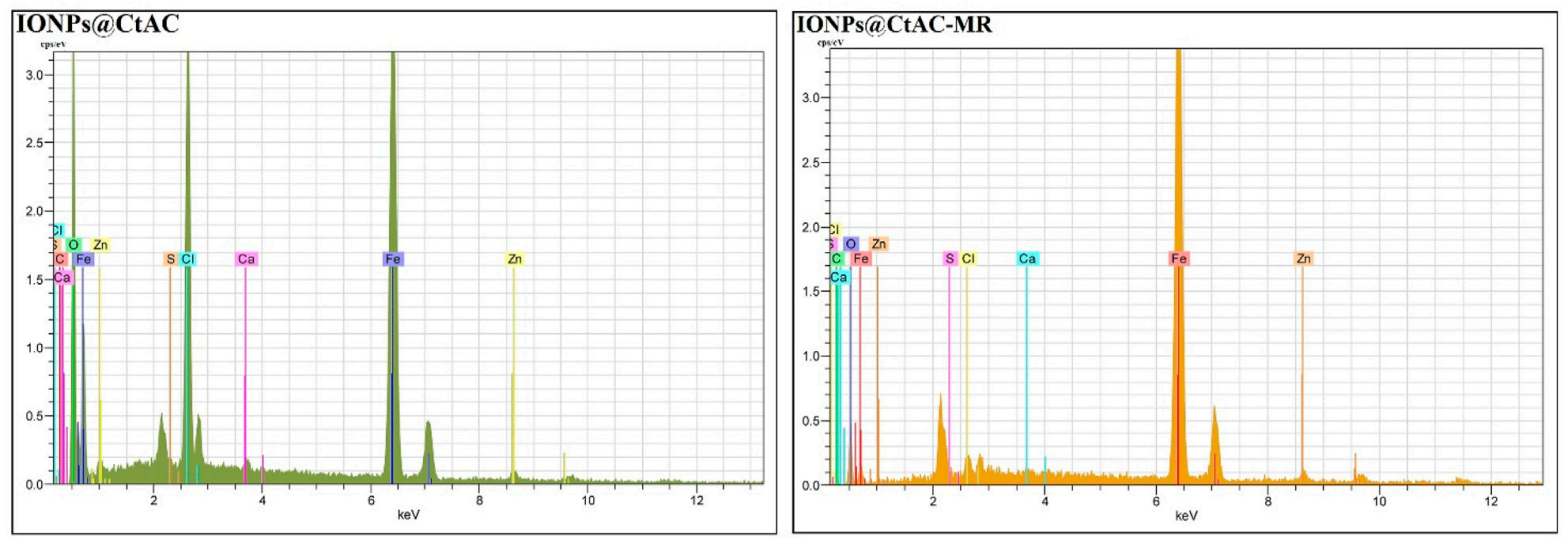
Energy-dispersive X-ray spectrum of IONPs@CtAC and IONPs@CtAC-MR.

The energy-dispersive X-ray (EDX) of IONPs@CtAC and IONPs@CtAC-MR magnetic nanocomposites is shown in Figure 4. The synthesized IONPs@CtAC magnetic nanocomposite has 35.23% Fe, 47.56% O, and 0.64% C elements, while the IONPs@CtAC-MR magnetic nanocomposite has 41.32% Fe, 48.98% O, and 2.17% C elements. When the EDX results were examined, it was seen that the IONPs@CtAC magnetic nanocomposite was successfully synthesized and the MR drug was successfully loaded into the IONPs@CtAC magnetic nanocomposite (Ferrone et al., 2022).

### 3.4 XRD analysis

**FIGURE 5:**
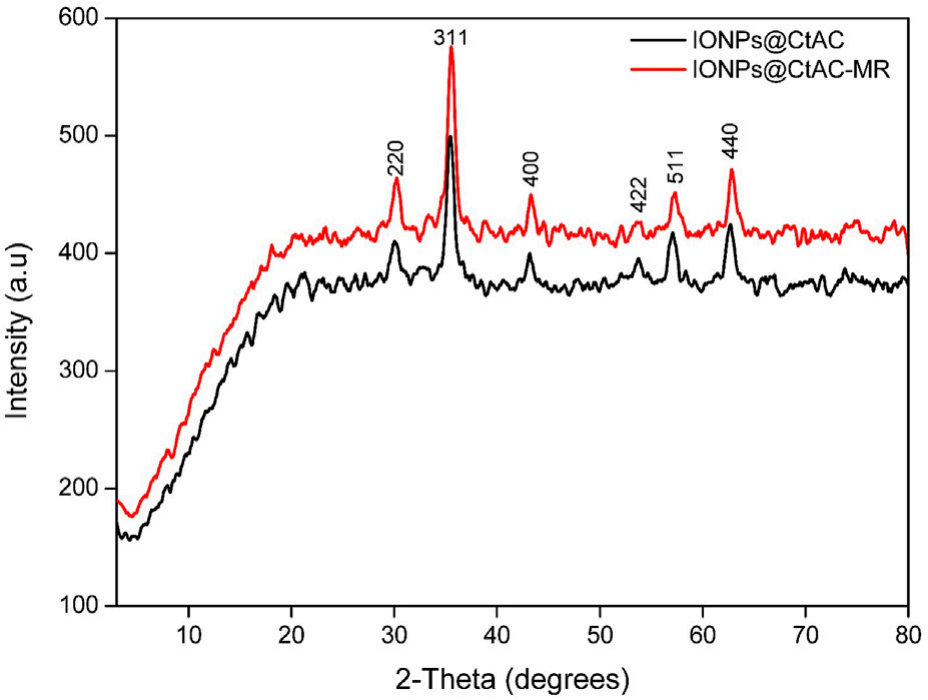
XRD patterns of IONPs@CtAC and IONPs@CtAC-MR.

Figure 5 shows the XRD patterns of IONPs@CtAC and IONPs@CtAC-MR. The diffraction peaks at (2*θ* = 30.35°, 35.660°, 43.40°, 53.75°, 57.50°, 64.85°, and 74.65°), which correspond to (220), (311), (400), (422), (511), and (440), can be easily indexed to a cubic spinel structure of magnetite according to the standard XRD pattern of IONPs@CtAC and IONPs@CtAC-MR. In this work, the Joint Committee on Powder Diffraction Standards (JCPDS) value for CtAC/MNP nanocomposite was 28-0491, and that for IONPs@CtAC-MR nanocomposites was 26-1136. According to the Scherrer equation, the crystal sizes of IONPs@CtAC and IONPs@CtAC-MR nanocomposites were 24.80 and 23.52 nm, respectively. XRD shows that IONPs@CtAC and IONPs@CtAC-MR nanocomposites were produced (Kerry et al., 2023).

### 3.5 VSM analysis

**FIGURE 6:**
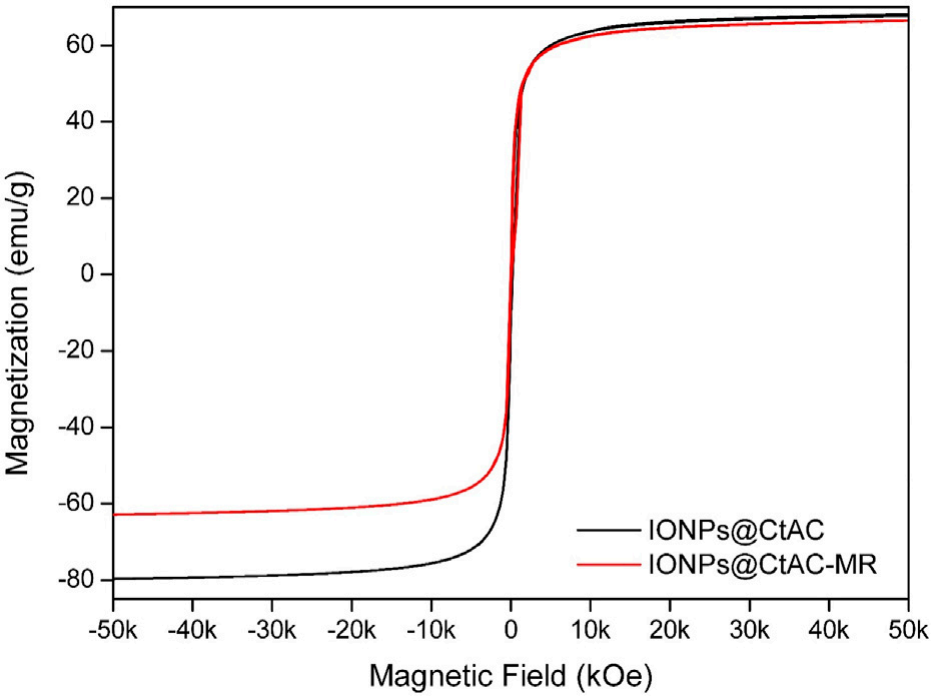
Magnetization curve of the IONPs@CtAC nanocomposite at 298 K.

Figure 6 shows the magnetization curves obtained by using the VSM technique to measure the magnetic behavior of IONPs@CtAC and IONPs@CtAC-MR nanocomposites. The superparamagnetic behavior of magnetic nanocomposites is indicated by the absence of hysteresis loop, coercivity, and zero remanence at ambient temperature. The saturation magnetization (Ms) of IONPs@CtAC and IONPs@CtAC-MR was measured to be 67.72 and 66.41 emu/g, respectively. The IONPs@CtAC magnetic nanocomposite showed a slight decrease in the Ms value after loading of MR compared to the IONPs@CtAC magnetic nanocomposite. The decreased Ms can be explained by the binding of MR in porous structures in magnetic nanocomposites (Bagherzadeh et al., 2023; Vinayagam et al., 2022; Nasaj et al., 2024). In addition, the morphological state of IONPs@CtAC and INPs@CtAC-MR can support saturation magnetization compared to other existing alternatives in the literature, such as cubic (Wang et al., 2020), rod-like shape (Marcuello et al., 2018), and nanoflowers (Theodosiou et al., 2022).

### 3.6 The zeta-potential measurements

**FIGURE 7:**
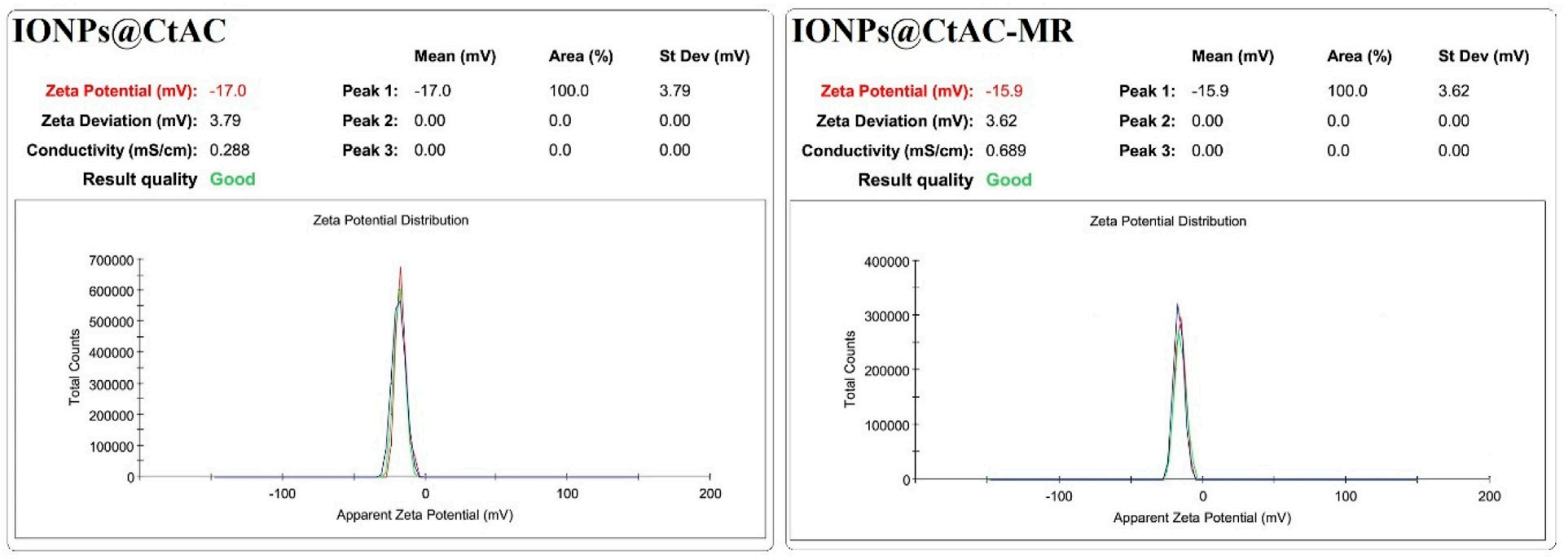
Zeta potential of the IONPs@CtAC nanocomposite and IONPs@CtAC-MR.

The zeta-potential measurements are presented in Figure 7, and the zeta potentials of the IONPs@CtAC nanocomposite and IONPs@CtAC-MR are negative. The result shows that both IONPs@CtAC nanocomposite (−17 mV) and IONPs@CtAC-MR (−15.9 mV) are negatively charged (Wang et al., 2023).

### 3.7 DLS analysis

**FIGURE 8:**
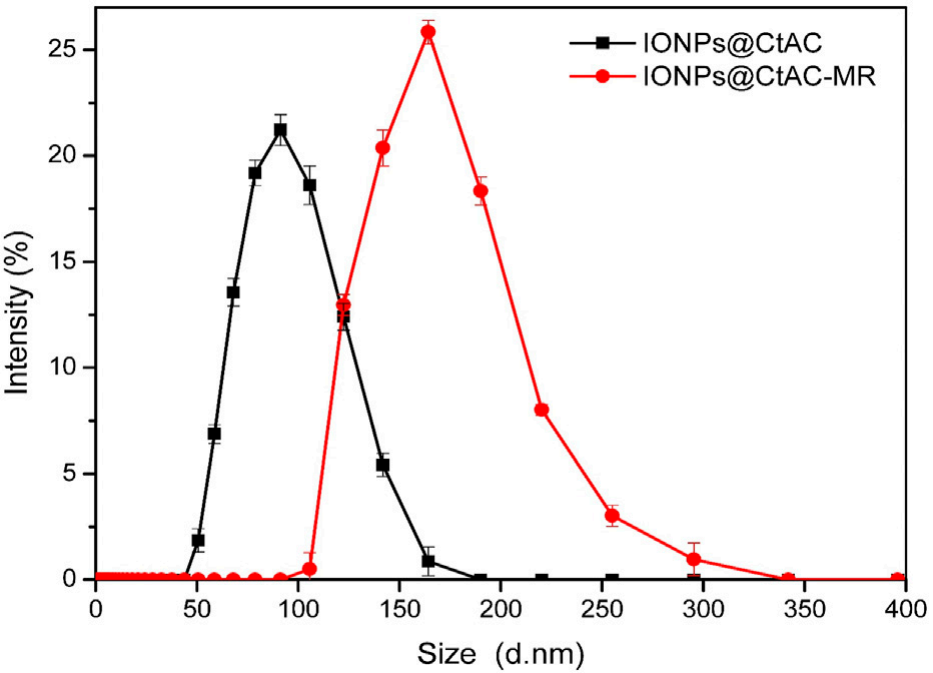
DLS analysis of IONPs@CtAC and IONPs@CtAC-MR.

According to the DLS data in Figure 8, it can be seen that the size of the IONPs@CtAC nanocomposite is 91.28 nm and the size of the IONPs@CtAC-MR is 164.183 nm (Mondal et al., 2022).

### 3.8 pH-dependent release behavior

**FIGURE 9:**
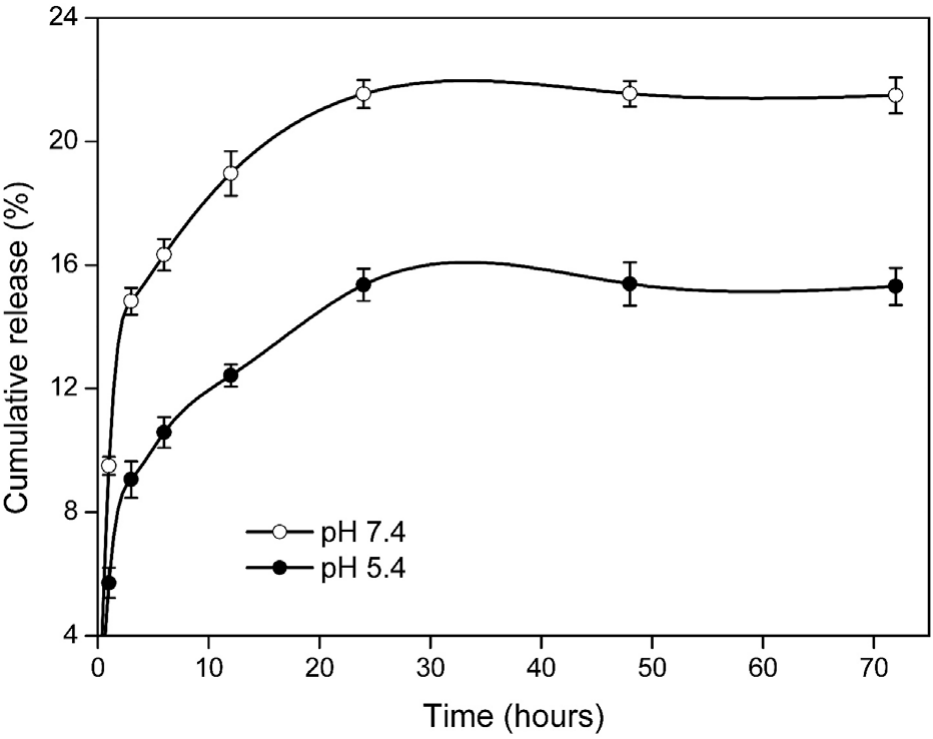
Cumulative release of IONPs@CtAC-MR at pH 7.4 and pH 5.4.

The cumulative release of MR loaded in IONPs@CtAC-MR at pH 7.4 and pH 5.4 prepared with phosphate buffer at 37°C is shown in Figure 9. In our study, the release of MR loaded on IONPs@CtAC showed a lower release in acidic conditions, where the release was pH-dependent (Cunha et al., 2023). To perform the release tests, 96 μg/mL MR loaded on IONPs@CtAC was used in 10 mL of the total release medium in different test tubes. For example, after 24 h, the release from IONPs@CtAC-MR with phosphate buffer at pH 7.4 was 20.17%, while it was 13.35% at pH 5.4. When the release of MR loaded on IONPs@CtAC was examined at pH 5.4, the release was measured as 10.58% after 6 h, 12.42% after 12 h, and 13.36% after 24 h (Kulkarni and Belgamwar, 2019; Jangid et al., 2020). When the release of MR loaded on IONPs@CtAC was examined at pH 7.4, it was detected as 13.73% after 6 h, 15.6% after 12 h, and 20.17% after 24 h (Jangid et al., 2018).

HT-29 colon (Thakur et al., 2024; Abbasi et al., 2023; Jain et al., 2023) and T98-G glioblastoma (Pinevich et al., 2022; Alkahtani et al., 2023) cell lines were used in different studies. HUVEC was used as a healthy cell line in this study. The cytotoxic effects of morin hydrate against HT-29, T98-G, and HUVEC cell lines in IONPs@CtAC were evaluated using the MTT assay (Lovato et al., 2024; Gomez-Gutierrez et al., 2021). Colorimetric tests are commonly used to assess the cellular metabolic activity, which is an indicator of toxicity (Pagar et al., 2022; Oh and Hong, 2022). The cell viability of HT-29, T98-G, and HUVEC cell lines and IONPs@CtAC-MR releases of pH 7.4 and 5.4 solutions prepared in phosphate buffer were evaluated after 24 and 48 h.

**FIGURE 10:**
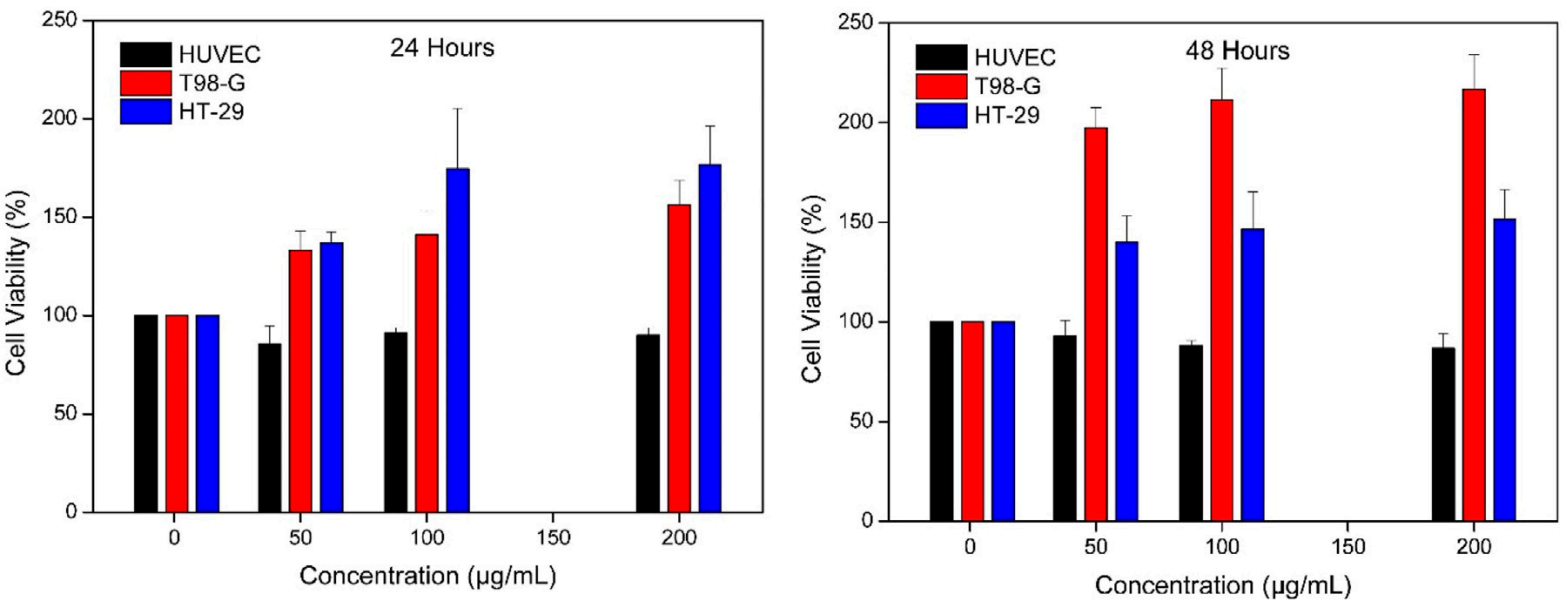
Effect of IONPs@CtAC-MR (1–200 μg/mL prepared with PBS 7.4 buffer) on the cell viability of HUVEC cell line, T98-G cell line, and HT-29 cell line. Cell viability was assessed using MTT assay after 24 and 48 h of exposure.

The 24-h exposure with MR-loaded IONPs@CtAC at pH 7.4 (Figure 10) did not cause significant differences in HT-29 and T98-G cells tested, and the 24-h cell viability was significantly above the range of IONPs@CtAC-MR concentrations tested.

**FIGURE 11:**
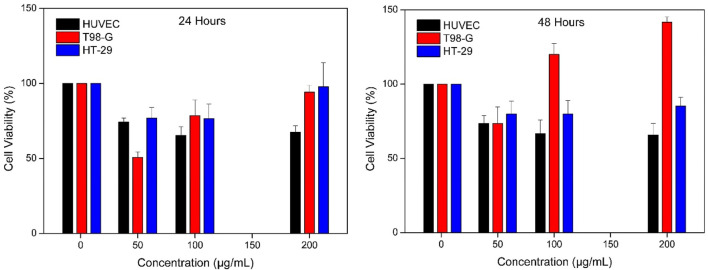
Effect of IONPs@CtAC-MR (1–200 μg/mL prepared with PBS 5.4 buffer) on the cell viability of the HUVEC cell line, T98-G cell line, and HT-29 cell line. Cell viability was assessed using MTT assay after 24 and 48 h of exposure.

However, 24-h exposure with MR-loaded IONPs@CtAC at pH 5.4 (Figure 11) caused significant differences in the HT-29 and T98-G cells tested, and the 24-h cell viability was significantly below the range of IONPs@CtAC-MR concentrations tested.

IONPs@CtAC-MR at 50 and 100 μg/mL reduced cell viability by 50% and 22%, respectively, in T98-G cells after 24 h of exposure, while it was the same for IONPs@CtAC-MR at 50 μg/mL after 48 h of exposure. IONPs@CtAC-MR reduced cell viability by 26% (Table 1). IONPs@CtAC-MR at 50 and 100 μg/mL reduced cell viability by 28.36% and 25.48%, respectively, after 24 h of exposure in HT-29 cells, while it was the same for IONPs@CtAC-MR at 50–100 and 200 μg/mL after 48 h of exposure. IONPs@CtAC-MR reduced the cell viability by 20.1% and 14.77%. According to the results in Table 1, it showed a proliferative effect in the T98-G cell line in 48 h. This strongly shows the wound healing effect of the material used in further studies. As a result, it is seen that the proliferative effect increases as the time increases.

**TABLE 1:** Measurement of cell viability with MTT in HT-29, T98-G, and HUVEC lines with IONPs@CtAC-MR

Cell Line	Concentration	Cell viability (%)			
		pH 7.4		pH 5.4	
		24 h	48 h	24 h	48 h
HUVEC	50 μg/mL	85.47 ± 9.30	92.82 ± 8.02	74.00 ± 2.68	73.51 ± 5.36
	100 μg/mL	91.12 ± 2.67	88.00 ± 2.58	65.43 ± 5.8	66.78 ± 8.94
	200 μg/mL	90.08 ± 3.74	86.74 ± 7.17	67.46 ± 4.38	65.65 ± 7.92
T98-G	50 μg/mL	133.27 ± 9.69	197.31 ± 57.31	50.72 ± 3.70	73.46 ± 11.17
	100 μg/mL	141.15 ± 12.10	211.32 ± 65.96	78.64 ± 20.43	120.04 ± 7.44
	200 μg/mL	156.20 ± 12.50	216.68 ± 87.44	94.25 ± 4.53	141.76 ± 3.49
HT-29	50 μg/mL	136.96 ± 5.67	139.97 ± 13.55	76.88 ± 7.32	79.90 ± 8.74
	100 μg/mL	174.56 ± 30.83	146.54 ± 18.69	76.62 ± 9.59	79.90 ± 9.01
	200 μg/mL	176.73 ± 19.43	151.49 ± 15.13	97.82 ± 15.91	85.23 ± 5.84

## 4 Conclusion

In this study, we synthesized the IONPs@CtAC magnetic nanocomposite for the delivery of morin hydrate, an anticancer drug. IONPs@CtAC was characterized by spectroscopic techniques such as FTIR, XRD, SEM-EDX, DLS, VSM, and zeta potential. Loading of morin hydrate (MR) onto IONPs@CtAC was carried out using the incubation technique. The loading efficiency of morin was measured as 96.0%. The encapsulation efficiency of MR provided a facilitating situation in release studies due to its effect of increasing both its solubility and dissolution rate, probably due to the transformation of the MR crystal into an amorphous form after loading into IONPs@CtAC. The increase in MR release at pH 5.4 and pH 7.4 suggests that IONPs@CtAC-MR may be a promising nanodelivery system to treat colon cancer (HT-29) and brain cancer (T98-G). IONPs@CtAC-MR showed concentration- and time-dependent cytotoxicity in T98-G glioblastoma cell lines and HT-29 colon cell lines. T98-G cells show a proliferative effect, which shows that the toxicity effect is low, and this may lead to the investigation of the wound healing effect of the IONPs@CtAC magnetic nanocomposite used in further studies. Moreover, the absence of any toxic effect can easily be carried to the target cell with this IONPs@CtAC magnetic nanocomposite in the T98-G cell line. Notably, the T98-G cell line showed more sensitivity to IONPs@CtAC-MR exposure treatments than HT-29 cells. In conclusion, our study suggests that magnetic IONPs@CtAC can be efficiently used as a targeted delivery mechanism for anticancer drugs with hydrophobic properties such as MR. Moreover, the above-synthesized nanomaterials may find application in various applied sciences. More *in vivo* models may provide more insights into the future use of these MR-loaded IONPs@CtACs and other similar materials.

## Data Availability

The original contributions presented in the study are included in the article/Supplementary Material; further inquiries can be directed to the corresponding authors.
